# Effects of Mechanical Stress Stimulation on Function and Expression Mechanism of Osteoblasts

**DOI:** 10.3389/fbioe.2022.830722

**Published:** 2022-02-17

**Authors:** Pan Liu, Ji Tu, Wenzhao Wang, Zheng Li, Yao Li, Xiaoping Yu, Zhengdong Zhang

**Affiliations:** ^1^ School of Clinical Medicine, Chengdu Medical College, Chengdu, China; ^2^ The First Affiliated Hospital of Chengdu Medical College, Chengdu, China; ^3^ Spine Labs, St. George & Sutherland Clinical School, University of New South Wales, Sydney, NSW, Australia; ^4^ Department of Orthopedics, West China Hospital of Sichuan University, Chengdu, China; ^5^ People’s Hospital of Jiulongpo District, Chongqing, China; ^6^ School of Public Health, Chengdu Medical College, Chengdu, China; ^7^ Basic Medical College of Chengdu University, Chengdu, China; ^8^ Department of Orthopedics, The First Affiliated Hospital of Chengdu Medical College, Chengdu, China

**Keywords:** mechanical stress, stimulation, function, expression mechanism, osteoblasts

## Abstract

Osteoclasts and osteoblasts play a major role in bone tissue homeostasis. The homeostasis and integrity of bone tissue are maintained by ensuring a balance between osteoclastic and osteogenic activities. The remodeling of bone tissue is a continuous ongoing process. Osteoclasts mainly play a role in bone resorption, whereas osteoblasts are mainly involved in bone remodeling processes, such as bone cell formation, mineralization, and secretion. These cell types balance and restrict each other to maintain bone tissue metabolism. Bone tissue is very sensitive to mechanical stress stimulation. Unloading and loading of mechanical stress are closely related to the differentiation and formation of osteoclasts and bone resorption function as well as the differentiation and formation of osteoblasts and bone formation function. Consequently, mechanical stress exerts an important influence on the bone microenvironment and bone metabolism. This review focuses on the effects of different forms of mechanical stress stimulation (including gravity, continuously compressive pressure, tensile strain, and fluid shear stress) on osteoclast and osteoblast function and expression mechanism. This article highlights the involvement of osteoclasts and osteoblasts in activating different mechanical transduction pathways and reports changings in their differentiation, formation, and functional mechanism induced by the application of different types of mechanical stress to bone tissue. This review could provide new ideas for further microscopic studies of bone health, disease, and tissue damage reconstruction.

## 1 Introduction

Mechanical forces affect almost every sphere of various life processes of living organisms, such as the perception of external hearing and touch, fluid flow and deformation during embryonic development, changes in cell osmotic pressure, pressure on blood vessel walls, and the movement of individual animals regulated by the earth’s gravitational environment. These forces range from mechanical stress signal generation, induction, and transduction to the final response, which involves the cell membrane, cytoderm, cytoskeleton, and other structures.

Bone tissue is very sensitive to mechanical stress stimulation. Unloading and loading of mechanical stress are closely involved in the differentiation and formation of osteoclasts and osteoblasts, and their bone resorption and formation functions, respectively (Robling and Turner, 2009; Li et al., 2020a). Consequently, mechanical stress exerts an important influence on the bone microenvironment and metabolism. Wolff’s Law points out that the lack of mechanical stress would lead to bone microstructure degeneration, mass loss and metabolism disorders, and would ultimately lead to osteoporosis (Brand, 2010). The absence of mechanical stress, such as with limb casts fixation, bed-rest, reduced exercise, and the weightlessness of astronauts in space, can lead to significant bone loss (Berg et al., 2007; Ragnarsson, 2015). In contrast, the mechanical load caused by exercise can restore bone mass and reverse these effects in most situations (Iura et al., 2015; Suniaga et al., 2018).

Exposure of tissues and cells to external mechanical stress transforms the external mechanical force into local mechanical signals in the body, triggering responses of cellular sensors. Subsequently, cellular mechanical signals are coupled to biochemical signaling molecules such as the nitric oxide produced and prostaglandins (PGs) (Duncan and Turner, 1995; Johnson et al., 1996; Klein-Nulend et al., 1997). Osteoblasts, osteocytes, bone lining cells, osteoclasts, and macrophages can sense mechanical stimulation and respond directly or indirectly (Dong et al., 2021). Mechanical transduction in bone tissue cells is a complex but precise regulatory process between cells and the microenvironment, between adjacent cells, and between mechanical sensors with different functions in a single cell. Ion channels, integrins, gap junction proteins, focal adhesion kinase, the extracellular matrix, the cellular skeletal components (such as intermediate filaments, microtubules, and actin filaments), and primary cilia are mechanical sensors that have been proven to regulate intracellular signaling pathways (Qin et al., 2020).

*In vitro* studies often use peripheral blood mononuclear cells, monocyte cells, bone marrow derived precursors and RAW264.7 cells induced to exhibit osteoclast formation (Owen and Reilly, 2018; Xiang et al., 2020). Bone mesenchymal stem cells (BMSCs), Human periodontal ligament cells (hPDLCs) and mouse embryo osteoblast precursor (MC3T3-E1) cells were induced to form osteoblasts (Rutkovskiy et al., 2016). The metabolic characteristics and mechanism of bone formation and remodeling have been explored by observing and studying the process of bone differentiation and formation. Osteocytes, osteoclasts and osteoblasts play major roles in bone tissue homeostasis. Bone tissue remodeling is a continuous process in which the role of osteoclasts is mainly in bone resorption, whereas that of osteoblasts is mainly bone remodeling, such as bone cell formation, mineralization, and secretion (Hardy and Fernandez-Patron, 2020). RANKL secreted by osteocytes binds to the receptor RANK on the precursor surface of osteoclasts to promote the differentiation and maturation of osteoclasts. Osteocytes also secrete OPG, which acts as the decoy receptor of RANKL and negatively regulates RANK signal to prevent osteoclast differentiation. When the ratio of RANKL/OPG increases, bone resorption increases; when the ratio of RANKL/OPG decreases, bone formation increases. In addition, osteocytes secrete sclerotin, which is a negative regulator of bone formation (Bonucci, 2009; Prideaux et al., 2016). These cell types balance and restrict each other to maintain bone tissue metabolism and homeostasis.

Previous studies have shown that appropriate mechanical stress stimulation can reduce the number and activity of osteoclasts and inhibit bone resorption, promote the differentiation and osteogenic function of osteoblasts, inhibit the differentiation of BMSCs into adipocytes, and prevent the loss of bone mass (Uzbekov et al., 2012). This effect also critically influences the regulation of bone metabolism signaling pathways (Uzbekov et al., 2012; Kameyama et al., 2013). However, the exact mechanism is not entirely clear. Numerous studies have further investigated the mechanism underlying the effects of mechanical stress on bone metabolism by examining mechanical stress stimulation in osteoclasts and osteoblasts (involving different species such as humans, mice and zebrafish,etc.) (Nomura and Takano-Yamamoto, 2000; Ho et al., 2005; Wittkowske et al., 2016). In this review, we review the mechanisms of mechanical stress stimulation on the function and expression of osteoblasts. Through this review, we attempt to provide a theoretical basis for the microscopic study of bone health, diseases, and injury reconstruction.

## 2 Mechanoreceptor

### 2.1 Ion Channels

Appropriate mechanical stimulation can activate calcium channels on the cell membrane to promote the transport of extracellular calcium into the cell, increasing the intracellular calcium concentration and promoting bone mass increase. Piezo1 and Piezo2 have been identified as important mechanosensitive channels. Piezo1 is a mechanosensitive ion channel through which osteoblasts sense and respond to changes in mechanical load and are required for gene expression changes caused by fluid shear stress (FSS) (Li et al., 2019; Zhou et al., 2020). Piezo1 expression in osteoblasts may also be promoted by mechanical tensile force (Wang et al., 2020a) and its deficiency in osteoblasts promotes bone resorption and contributes to osteoporosis in mice, but does not affect bone mass (Wang et al., 2020b).

Li et al. (Li et al., 2019) reported that the removal of Piezo1 from osteoblasts and bone cells does not completely eliminate the response of bones to mechanical stimuli. Furthermore, Piezo1 is not the only mechanosensor in osteoblasts and bone cells. Although Piezo1 and Piezo2 mRNA expression was detected in bone tissues, Piezo1 expression was significantly higher in bone cells and osteoblasts than that of Piezo2 (Sugisawa et al., 2020). Piezo2 is more involved in the development of the nervous system and the perception of touch and pain, than Piezo 1 is, including through molecules such as Merkel cells, outer hair cells and somatosensory ganglia (Woo et al., 2014). Other channels, such as the transient receptor potential (TRP) vanilloid (TRPV) and certain members of the epithelial Na^+^ channel (ENaC) protein family, can guide cation influx under a hypertonic environment or membrane tension, converting mechanical force signals into electrical and chemical signals (Gu and Gu, 2014; Kefauver et al., 2020).

### 2.2 Cytoskeleton

The cytoskeleton is a network structure in cells that is mainly composed of protein fiber (Kounakis and Tavernarakis, 2019), and mainly consists of microtubules, actin fibers, and intermediate filaments (Sandbo et al., 2016). It plays an important role in maintaining cell morphology, bearing external forces, and maintaining the internal cellular structure. The cytoskeletal structure is highly nonlinear, enabling cells to sense deformation and change and the complete cytoskeleton contributes to maintaining tight adhesion between cells and the extracellular matrix (ECM). The integrin glycoprotein family located on the cell membrane senses mechanical signals through interactions between the ECM and intracellular signals (Aisha et al., 2015). Human MSCs (hMSCs) can be gradually remodeled through cell recombination and arrangement and the regulation of smooth muscle cells by the cytoskeleton (Parandakh et al., 2017). Microtubule actin cross-linking factor 1 (MACF1) is a regulator of cytoskeletal dynamics that is necessary for maintaining bone tissue integrity (Wang et al., 2021a), whereas actin in the cytoskeleton is mainly involved in mechanical stress (Zhou et al., 2018). The link between mechanical stimulation, integrin/cytoskeleton/Scr/extracellular signal-regulated protein kinase (ERK) signaling pathway activation, and osteocyte survival provides a mechanical basis for the role of mechanical forces in the bone (Plotkin et al., 2005).

### 2.3 Integrin

Integrins are heterodimers formed by the non-covalent binding of *α* and *β* subunits and presently, mammals are known to express18 *α* and 8 *β* subunits, which combine to form 24 integrins (Selvakumarasamy et al., 2019). Integrin senses physical or biochemical stimulation of the ECM by binding to its ligands (including fibonectin, collagen, and laminin) in the extracellular region. Furthermore, through conformational changes, integrin mediates the transmission of signals to cells to induce their adhesion, migration, proliferation, and differentiation. Moreover, intracellular signal changes also affect the conformational changes of integrin, alter the affinity of ligand binding, and affect the biological behavior of cells. This integrin-dependent bidirectional signal transduction mechanism plays an important role in bone remodeling (Uda et al., 2017; Kong et al., 2020; Michael and Parsons, 2020). Recent studies have shown that osteoclasts express integrin *α*2 and *α*V and play an important role in bone resorption (Kong et al., 2020). Subsequent studies should focus on further clarifying the important role of integrin in mechanical stimulation and bone metabolism.

### 2.4 Primary Cilia

Primary cilia is widely found in osteocytes, MC3T3-E1 cells, murine long bone osteocyte-Y4 (MLO-Y4) osteoid cells, cranial osteoblasts, and hMSCs, and it is an important mechanoreceptor that responds to mechanical stimulation and coordinated load induction in these cells (Xiao et al., 2006; Malone et al., 2007; Hoey et al., 2012). However, the existence of primary cilia in osteoclasts has not been reported in existing studies (Yuan et al., 2015). The exposure of osteoblasts and hMSCs to suitable FSS upregulated the expression levels of runt-related transcription factor 2 (RUNX2), bone morphogenetic protein 2 (BMP2), alkaline phosphatase (ALP), and osteopontin (Sonam et al., 2016). In bone cells, primary cilia act as mechanosensors that respond to and flex extracellular fluid impulses generated by the body during walking and running. When primary cilia bend, the increased tension on the membrane opens mechanosensitive ion channels, which leads to intracellular Ca^2+^ influx, membrane depolarization, and activation of nerve fibers and then, the cell experiences mechanical stimulation (Whitfield, 2003). Studies have shown that a simulated microgravity environment eliminates the formation of primary cilia, inhibits the formation and mineralization of rat skull osteoblasts, and significantly shortens the residual cilia (Shi et al., 2020). However, the specific mechanism underlying the action of primary cilia in the bone under mechanical stress has cannot be fully explained by current studies, and further elucidation is needed in future studies.

## 3 Mechanical Stimulation With Osteoblasts

### 3.1 Gravity

The force through which objects are attracted to each other on earth is called gravity, which is exerted by the earth, and the direction of gravity is always straight down.

#### 3.1.1 Hypergravity and Osteoblasts

MC3T3-E1 cells as osteoblast precursors are commonly used for mechanical sensing and gravity studies, which often use different methods or stimulatory interventions to observe the effects of osteoblast differentiation. Hypergravity (a force of 5, 10, 20, and 40 × g) was shown to promote the proliferation of MC3T3-E1 and osteoblast-like cells through a PGE2-mediated mechanism *in vitro* (Miwa et al., 1991), increase ALP activity, and was positively correlated with the duration of hypergravity (Nakajima, 1991). Similarly, hypergravity of 3 × g stimulates bone formation by enhancing the activity of osteopontin and RUNX2 in osteoblasts (Zhou et al., 2015). Kawao et al. (Kawao et al., 2020) found increased mRNA levels of RUNX2, osterix, ALP, and osteocalcin; ALP activity; and mineralization *in vivo* in osteoblasts from mice exposed to 3 ×g hypergravity. In addition, Woodcock et al. (Woodcock et al., 2019) found that high gravity effectively increased the intracellular viscosity of MC3T3-E1 cells and promoted the maturation and differentiation of osteoblasts, whereas higher levels (10, 15, and 20 × g) had a more significant effect.

#### 3.1.2 Microgravity

The force of gravity in space is one millionth that on earth (9.8 m/s^2^). Microgravity can lead to osteoblast and osteoclast interaction disorders, resulting in bone loss, muscle relaxation, and the development of osteoporosis. Space microgravity and simulated microgravity (such as in plaster splintage, tail suspension test, sciatic denervation, and hindlimb unloading are commonly used in studies of bone metabolism *in vivo* and *in vitro* (Globus and Morey-Holton, 2016).

##### 3.1.2.1 Microgravity and Osteoblasts

In space microgravity and simulated microgravity, sclerostin (SOST) enhances osteoclast formation by decreasing the production of osteoprotegerin (OPG) in osteoblasts and increasing the secretion of receptor activator of nuclear factor (NF)-κB ligand (RANKL) (Saxena et al., 2011; Chatani et al., 2016). Similar studies have shown that microgravity reduced osteoblast production and enhanced that of osteoclasts by decreasing OPG secretion by osteoblasts (increasing the RANKL/OPG ratio) (Rucci et al., 2007). Serum glucocorticoid levels increased significantly on day 3 in an animal model of hindlimb unloading that simulated microgravity. High glucocorticoid levels inhibited the expression of Wnt/*β*-catenin signaling pathway molecules and upregulated the expression of SOST in bone cells (Yang et al., 2020). Therefore, enhanced secretion of glucocorticoid may be an important factor for bone loss in the hindlimb unloading model (Yang et al., 2020). A recent study showed that the leukemia inhibitory factor (LIF) enhanced signal transducer and activator of transcription 3 (STAT3) phosphorylation in BMSCs and increased the expression of ALP and osteogenic genes. However, in the *in vitro* microgravity environment, the secretion of LIF by bone cells was inhibited, which weakened the osteogenic effect (Du et al., 2020). Microgravity also increases the level of oxidative damage markers in the body and weakens the total antioxidant capacity.

Reactive oxygen species (ROS) inhibit the function of osteoblasts (Manolagas, 2010; Steller et al., 2018), promote MC3T3-E1 cell apoptosis, and downregulate the expression of MAF BZIP transcription factor G (MafG) (Wang et al., 2021b). Therefore, increased ROS expression in microgravity is also one of the key factors for bone loss. In addition, microRNA (miR)-494 inhibits BMP2-induced osteoblast differentiation by downregulating BMPR2 and RUNX2 under microgravity simulation (Qin et al., 2019). An increasing number of studies have shown that the inhibition of osteogenic differentiation in microgravity can be regulated by multiple non-coding RNAs (ncRNAs) (Wang et al., 2018; Wang et al., 2020c; Cao et al., 2021).

Although resistance training for astronauts can effectively prevent bone loss (Stein, 2013), the adverse effects of the space microgravity environment on the bones of astronauts can last for several years, which highlights the importance of studying bone metabolism under microgravity conditions. The huge cost of *in vivo* and *in vitro* experiments on space flight has necessitated the development of a variety of experimental platforms to simulate ground microgravity environments (Bonnefoy et al., 2021; Iordachescu et al., 2021). However, fully replicating the changes induced by space microgravity exposure is challenging and, therefore, the develop of more reliable and accurate ground microgravity simulation environments for in-depth research is necessary.

#### 3.1.3 Continuously Applied Compressive Pressure

The force perpendicular to the surface of a fluid per unit area is called the static pressure of the fluid, and continuous action for a specified period of time is called the continuously applied compressive pressure (CCP) (Xing et al., 2004). As a form of stress stimulated by mechanical stress, CCP is an effective condition for stimulating bone tissue growth. However, when CCP is too high or insufficient, it can reduce bone mass and lead to bone loss.

##### 3.1.3.1 Continuously Applied Compressive Pressure and Osteoblasts

Imamura et al. (Imamura et al., 1990) Found that CCP inhibits osteoblast differentiation of MC3T3-E1 cells through production of PGE2. Their further study found that applying continuous static pressure (3 atm, ATM) to MC3T3-E1 cells inhibited the ALP activity of the osteoblasts and promoted PGE2 secretion. When MC3T3-E1 cells were transferred to a CO_2_ incubator at 1 ATM, the inhibition of ALP activity was rapidly reversed (Ozawa et al., 1990). Yanagisawa et al. (Yanagisawa et al., 2008) reported that 1.0 g/cm^2^ compressive stress was the optimal condition for osteoblast differentiation, and the study by Tripuwabhrut et al. (Tripuwabhrut et al., 2013) showed that osteoblast differentiation was enhanced when compressive stress increased from 2.0 g/cm^2^ to 4.0 g/cm^2^. Xiaoqing Shen et al. (Shen et al., 2017) showed that compressive stress at the range of 5.0 g/cm^2^ had no significantly different effects on the survival rate of MC3T3-E1 cells.

These results indicate that CCP has a positive effect on the differentiation of MC3T3-E1 cells and osteoblasts, but the exact dose of CCP for MC3T3-E1 cells and specific effects are currently unclear. Recently, Shu et al. (Somemura et al., 2021) reported that treating osteoblasts in a three-dimensional (3D) cell-collagen sponge construct with 25.5 gf/cm^2^ (2.5 kPa) for 24 h upregulated glucose transporter1 (Glut1), RUNX2 and ALP, whereas silent mating type information regulation 2 homolog 1 (sirtuin 1, SIRT1) was downregulated in osteoblasts induced by compressive mechanical loading. They hypothesized that the Glut1/SIRT1/RUNX2 pathway in osteoblasts may play a role in mechanical stress-induced bone formation and osteoblast differentiation. *In vitro* studies have repeatedly confirmed that osteocytes (not only osteoblasts, but also osteoblasts, osteoclasts and their progenitors) do indeed exhibit altered activity at hydrostatic pressures of up to 1 Hz, with amplitudes of tens (to hundreds) of kilopascals. Since *in-situ* tests are somewhat difficult to achieve, Whether hydrostatic pressure, identified *in vitro* as mechanical stimulation, actually occurs *in vivo* is controversial (Duncan and Turner, 1995). With this problem in mind, the multi-scale mechanical biology method and multi-scale mechanical model proposed by Scheiner et al. that connect porous micromechanics and mathematical systems biology provide valuable insights for this problem (Scheiner et al., 2014; Scheiner et al., 2016; Estermann and Scheiner, 2018). In addition, reports of CCP on osteoblast differentiation and osteoclast differentiation regulated by osteoblasts are still being explored (Tripuwabhrut et al., 2013). The effects of CCP strength and duration on osteoblasts will be precisely defined in future studies.

#### 3.1.4 Tensile Strain

Various types of stress can produce relative strain when applied to an object. The ratio of the length, shape, and volume variation of the object before and after the action of tensile stress (single/bidirectional tensile stress) is called tensile strain. Ilizarov (Ilizarov, 1989) was the first to successfully apply traction stress to stimulate bone formation, and proposed the theory of “distraction osteogenesis”. To date, the theory has been successfully applied in strategies for the repair and treatment of bone defects (Kani et al., 2020).

##### 3.1.4.1 Tensile Strain and Osteoblasts

The tensile stress of osteoblasts cultured *in vitro* was mainly achieved by stretching the culture medium membrane that adhered to the osteoblasts. In applying stretch stress to the culture medium membrane, the strength and duration can be controlled to observe the different responses of osteoblasts to stretch tension under different conditions. Furthermore, this process passively pulls osteoblasts that have adhered to the culture medium membrane, which simulates osteoblast stress *in vivo*. Chen et al. (Chen et al., 2018) found that mechanical stretching of human jaw bone marrow MSCs using the Flexcell tension system *in vitro* significantly increased ALP activity and calcium deposition. Moreover, the expression levels of RUNX2 and osterix were significantly upregulated, whereas NF-кB was significantly downregulated. Davidson et al. (Davidson et al., 2013) performed a mandibular osteotomy and pull experiment on Sprague-Dawley rats using a pull osteogenesis technique. The results showed that BMP2, intranuclear SMAD family member 1 (SMAD1) phosphorylation and ALP activity were increased in the traction region, and the osteogenic effect was significantly increased. Wnt1 expression was found to be elevated in alveolar bone cells on the tension side of orthodontic tooth movement (OTM) mouse model for 5 days (Ei Hsu Hlaing et al., 2020).

In addition to mechanical tensile force acting directly on osteoblast precursors and osteoblasts, it can also induce osteogenesis indirectly by affecting other mechanosensitive cells. Dong et al. (Dong et al., 2021) reported that macrophages are exposed to cyclic stretching with a 5% strain at a frequency of 1.0 Hz for up to 12 h using the FX-4000 Flexcell, and the stretched macrophages were co-cultured with BMSCs. The expression of opsin (OPN) and RUNX2 was significantly increased and induced Yes-associated protein (YAP) activation and nuclear translocation, which subsequently regulated downstream BMP2 expression to promote BMSCs osteogenesis (Dong et al., 2021). Li et al.(2020b) induced distraction osteogenesis of osteoblasts, and found that their proliferation was enhanced and mRNA levels of ALP, RUNX2, osteocalcin (OCN), collagen type I, hypoxia-inducible factor (HIF)-1*α* and vascular endothelial growth factor (VEGF) were significantly increased.

However, *in vivo* studies showed that locally generated bacterial inflammation inhibited RUNX2 expression and up-regulated c-fos and interleukin (IL)-1*β* expression levels in osteoblasts induced by tensile strain, and reduced the osteogenesis of osteoblasts under continuous tensile stress (Lacey et al., 2009; Li et al., 2021). Obesity induced by high-fat diet decreases osteoblast activity in alveolar bone of the OTM stretching side (Luo et al., 2021). Therefore, it is necessary to consider several factors to maximize the function of osteoblasts using tensile stress.

#### 3.1.5 Fluid Shear Stress

Fluid shear stress (FSS) is a type of mechanical stress caused by extracellular fluid, such as tissue fluid, flowing through the cell membrane surface, and the theory that FSS is induced by the flow of tissue fluid in the lacunar-canalicular system is widely accepted (Kumar et al., 2021). Applying loads (including mechanical loads, muscle contractions, blood pressure, and lymphatic drainage) to the bones causes the interstitial fluid to flow, which compresses the lacunar-canalicular system, thereby inducing various mechanical stimuli including FSS (Duncan and Turner, 1995; Tanaka et al., 2005; Price et al., 2011). FSS can further induce changes in the biomechanical properties of osteoblasts.

#### 3.1.6 Fluid Shear Stress and Osteoblasts

Previous studies have shown that FSS inhibits tumor necrosis factor (TNF)-*α*-induced osteoblast apoptosis (Pavalko et al., 2003). More recent studies have shown that FSS can activate the ERK5-serine-threonine protein kinase B (AKT)-forkhead box O3a (FoxO3a)-Bim/FasL signaling pathway, inhibit the activation of caspase 3, and protect osteoblasts from apoptosis induced by TNF-*α* (Bin et al., 2016). Wang X. et al. (Wang et al., 2021e) found that the expression level of long-coding (RNA) lncRNA taurine up-regulated 1 (TUG1) increased in a time-dependent manner when MC3T3-E1 cells were exposed to 12 dyn/cm^2^ FSS treatment for 30, 60, and 90 min. LncRNA TUG1 upregulated fibroblast growth factor receptor 1 (FGFR1) expression by sponging miR-34a, which promoted osteoblast proliferation and inhibited osteoblast apoptosis (Wang et al., 2021e).

This study also found that FSS downregulated miR-140-5p and promote osteoblast proliferation by activating the vascular endothelial growth factor-A (VEGFA)/ERK5 signaling pathway (Wang et al., 2021d). FSS increased the expression of Piezo1 in MC3T3-E1 cells, activated the AKT- serine-threonine protein kinase glycogen synthase kinase 3 (GSK3)/*β*-catenin pathway and upregulated the expression level of RUNX-2 (Song et al., 2020). FSS remodels the cytoskeleton of MC3T3-E1 cell; arranges F-actin proteins in one direction, making them more compact and uniform; and increases the expression level of phospho-paxillin and integrin-*α*5 (Jin et al., 2020).

Recently, the in-depth study of bone ingrowth between internal fixation materials and bone interface has encouraged the study of material-bone interface-mechanical stimulation as an emerging focus. Lei et al.(2021) reported the effect of FSS on human MG-63 osteoblast-like cells on titanium with different surface modifications, which was particularly evident on the implant-bone interface. This study found that FSS (12 dyn/cm^2^) significantly induced cell proliferation and upregulated the expression level of focal adhesion kinase (FAK), which the authors speculated that FAK may play a key role in the mechanical transduction of the implant-bone interface (Lei et al., 2021).

FAK is a nonreceptor tyrosine kinase, which plays a key role in integrin-mediated signal transduction and downstream signaling pathways (Tapial Martinez et al., 2020). Wang J. et al. (Wang J. et al., 2021) found that the interface force or adsorption force of protein-material can regulate the organization of the actin cytoskeleton and promote the formation of focal adhesion, and that FSS promotes assembly of the actin cytoskeleton and decomposition of focal adhesions. These emerging studies will contribute to the designing of a harmonious bioreactor and mechanical load to facilitate the comprehensive study of bone tissue regeneration.

## 4 Signal Pathways Mediating Effects of Mechanical Stress Stimulation on Osteoblasts

Mechanical load induces stress stimulation of the bone and transmits the force to the bone cells. The receptors on the cell membrane are activated by binding of specific ligands, which triggers the signal cascade and enhances expression of downstream target genes, playing a role in bone metabolism. Mechanical transduction is a complex process regulated by multiple signal pathways.

### 4.1 Wnt/*β*-Catenin Signaling Pathway

The Wnt/*β*-catenin pathway is one of the most important pathways studied in bone metabolism research. Exposure of the transmembrane co-receptors low-density lipoprotein (LDL) receptor related protein 5 (Lrp5), Lrp6, and Frizzled (FZD) protein family to Wnt, activates the Lrp/FZD receptor complex on the cell surface, which inhibits the degradation activity of *β*-Catenin through the phosphorylation of downstream protein kinases. Subsequently, the stably accumulated *β*-catenin in the cytoplasm enters the nucleus and combines with the T-cell factor/lymphoid enhancer factor (TCF/LEF) transcription factor family to initiate the transcription of downstream target genes to promote the expression of osteogenic genes (such as *OPG*) and promote bone formation. Studies have found that mice with Lrp5 deletion mutations continue to show a state of low bone mass (Sawakami et al., 2006; Iwaniec et al., 2007).

More recent studies showed that human patients with Lrp5 missense mutation (A745V) had severe osteoporosis, which may be attributable to the weakening of the anabolic response of bones to mechanical stress (Kiel et al., 2007; Norwitz et al., 2019). Lrp5 and Lrp6 have been shown to be extremely important in bone cell mechanical transduction (Jing et al., 2016). In addition, under mechanical load, heterozygous deletion of *β*-catenin in a single osteocyte in mice eliminates the bone synthesis response to mechanical load and the ability to form new bone (Javaheri et al., 2014). Numerous studies using *in vitro* and *in vitro* models have also demonstrated the involvement of the Wnt pathway in mechanical stress (Bonewald and Johnson, 2008; Duan and Bonewald, 2016; Kang et al., 2016; Fu et al., 2020). However, SOST negatively regulates the Wnt/*β* -catenin pathway (Choi and Robling, 2021).

Dickkopf-related protein (DKK) 1 and DKK2 are expressed by osteocytes and negatively regulate Wnt/*β*-catenin. The mechanical load can inhibit the expression of DKK1 in osteocytes and promote the upregulation of Wnt signaling (Holguin et al., 2016). *In vivo* studies using hypergravity (3 × g) showed significantly reduced DKK2 levels in the serum and DKK2 mRNA levels in the soleus muscle of mice (Kawao et al., 2020). DKK2 was shown to inhibit mRNA levels of RUNX2, osterix, ALP, and osteocalcin and alkaline phosphatase activity and mineralization in osteoblasts, and enhance the phosphorylation of *β*-catenin in mouse osteoblasts (Kawao et al., 2020). Regulation of the Wnt pathway has a positive effect on compensatory mechanisms for coping with mechanical stress and changing bone mass, but the specific mechanism remains to be further studied.

### 4.2 Notch Signaling Pathway

The Notch signaling pathway, which is expressed in almost all organ systems, is highly conserved and plays an important role in the occurrence and development of diseases by regulating various cell processes (Andersson et al., 2011). Mammals have multiple Notch receptors, and the common types are Notch 1, Notch 2, Notch 3, and Notch-4. The Notch receptor is a one-way transmembrane protein that consists of the Notch extracellular domain (NECD), Notch transmembrane domain (NTM), and Notch intracellular domain (NICD). In mammalian cells, members of the delta-like ligand (DLL1, DLL3, and DLL4) and Jagged (JAG1 and JAG2) families act as ligands for Notch signaling receptors (Steinbuck and Winandy, 2018). Binding of the specific ligand to the receptor activates the Notch signaling pathway and its downstream target genes [including hes family bHLH transcription factor 1 (*Hes1*), hes related family bHLH transcription factor with YRPW motif 1 (*Hey1*), and *Hey2*] are transcribed. It is worth mentioning that Hes1 is a key factor in bone metabolism (Zanotti et al., 2011).

The role of Notch receptors in bone metabolism has always been controversial. Overexpression of NICD1 in osteocytes reduces bone resorption, leading to an increase in bone mass. Conversely, specific activation of Notch signaling in immature osteoblasts impaired their differentiation, leading to osteopenia (Canalis et al., 2013). Wang L. et al. (Wang et al., 2020a) found that mechanical stretch stress can activate the Notch1 signaling pathway and the expression of ALP, RUNX2, OCN, bone sialoprotein (BSP), and promote the osteogenic differentiation of human periodontal ligament stem cells. Ziouti et al. (Ziouti et al., 2019) found that applying tibial cyclic compression load (216 cycles at 4 Hz, peak strains at a tibial midshaft of +900 *με*) to wild-type mice induced specific Notch target genes (mRNA expression of Hes1, Hey1, and Hey2). Cyclic stretching of primary human BMSCs increased the gene expression of Notch receptors *Notch1* and *Notch2* by more than 60-fold and 30-fold, respectively. RUNX2 and mechanical response genes prostaglandin-endoperoxide synthase 2 (*PTGS2*) and *FOS* were upregulated. Studies have shown that the Notch pathway is activated and osteogenic gene expression is upregulated in osteoblasts under mechanical stress. However, the exact mechanism of the Notch pathway-related effects in osteoblasts under mechanical stress has not been fully elucidated.

### 4.3 ERK5 Signaling Pathway

ERK, which belongs to the mitogen-activated protein kinases (MAPKs) family, has strong catalytic activity and plays a key role in upstream signal transduction pathways including mechanical signals in cellular reactions (Fey et al., 2012; Yuan et al., 2020). Studies have shown that loading MC3T3-E1 cells with FSS increases cyclooxygenase (COX)-2 activity, which activates osteoblast-like cell proliferation and anabolic metabolism (Bo et al., 2016; Ding et al., 2019). Knockout of ERK5 using small interfering RNA (siRNA) prevented FSS from upregulating COX-2 and cyclic adenosine monophosphate (cAMP) (Jiang et al., 2015). Wang X. et al. found that FSS-induced downregulation of miR-140-5p regulates ERK5 signal activation through VEGFA and promotes osteoblast proliferation (Wang et al., 2021d). FSS inhibits caspase 3 activation to prevent osteoblast apoptosis by activating the ERK5-AKT-Foxo3a pathway in MC3T3-E1 cells (Bin et al., 2016). Other studies have shown that the effect of mechanical load on ERK5 is an important factor affecting the proliferation of osteoblasts (Li et al., 2012; Zhang et al., 2021).

### 4.4 RhoA Signaling Pathway

RhoA belongs to the Rho Family of small GTPases, and is a key regulator of actin cytoskeleton. The mammalian genome encodes about 20 kinds of Rho gtpase, and currently the most researched ones are RhoA, Rac1 and Cdc42 (Ridley and Hall, 1992). The activation and inactivation of RhoA is regulated by signals from intracellular and extracellular G protein coupled receptors, integrins and growth factor receptors (Kaneko-Kawano and Suzuki, 2015). Members of the Rho associated protein kinase (ROCK) family, including ROCK1 and ROCK2, are important effectors of RhoA (Arnsdorf et al., 2009). When exogenous forces or forces generated by the cell itself and the cytoskeleton act on the cell (Chen, 2008), cell surface adhesions, cytoskeleton and membrane tension work together to affect mechanosensors and stimulate mechanical responses (Vogel and Sheetz, 2006; Hoffman et al., 2011). Studies have shown that Rho/ROCK converts mechanical stimulation into gene expression changes through the actin-myocardin-related transcription factor (MRTF)-serum response factor (SRF) pathway (Zhao et al., 2007). Dupont et al. (Dupont et al., 2011) found that stretch stimulation can activate the RhoA/ROCK signaling pathway and YAP/transcriptional co-activator with PDZ-binding motif (TAZ), promote bone formation, inhibit adipogenesis, and form actin at the same time. Similarly, RhoA feels high ECM stiffness through focal adhesion, promotes actin polymerization and stress fiber formation, transmits stiffness signals to YAP/TAZ, and regulates the sensitivity of osteoblasts (Wagh et al., 2021). MRTF and YAP/TAZ have been confirmed as transcription factors activated by mechanical induction (Finch-Edmondson and Sudol, 2016; Cai et al., 2021). The activity of ROCK is positively correlated with ECM stiffness. Although stiffness sensing is related to the regulation of multiple signal transductions, RhoA/ROCK pathway is perhaps the most prominent (Selig et al., 2020). Studies have been found that RhoA and its effector protein ROCKII regulate the differentiation of C3H10T1/2 cells induced by oscillatory fluid flow to osteogenic differentiation. At the same time, activated RhoA and fluid flow have an additive effect on the expression of RUNX2 (Arnsdorf et al., 2009). Gardinier et al. (Gardinier et al., 2014) has shown that under the influence of FSS, the mechanical sensitivity of MC3T3-E1 cells is regulated by the activation of P2Y_2_ receptors through the RhoA/ROCK signal cascade under the influence of FSS. The RhoA signalling pathway is central to mechanotransduction because it plays a key role in regulating the catin cytoskeleton and its response to mechanical force.

In addition, other signaling pathways such as the transforming growth factor-*β* (TGF*β*)-Smad (Zou et al., 2021), NF-κB (Wang et al., 2015), and BMP(da Silva Madaleno et al., 2020; Wei et al., 2020), signaling pathways also have an important role in the responses of osteoblast-like cells under mechanical stimulation, and there is crosstalk between various signaling pathways (Kopf et al., 2014; Grafe et al., 2018; Shuai et al., 2018). Moreover, the effects of mechanical stimulation on the various signal pathways mediated the response of osteoblasts are interactive and related (Table 1). Once mechanical stimulus receptors are affected by mechanical loading and unloading, it is possible to activate multiple biological signaling pathways during the conversion of physical signals into chemical stimulation signals, thus generates positive or negative regulation of osteoblast cells. However, the current research is not thorough, and its mechanism needs to be further elucidated. Future research should continue to focus on clarifying the signal pathways mediating the mechanism underlying the actions of mechanical stimulation on osteoblast-like cells.

**TABLE 1:** Signal Pathways Mediating Effects of Mechanical Stress Stimulation on Osteoblast like cells. FSS, fluid shear stress; OPG: osteoprotegerin; DKK, Dickkopf-related protein; RUNX2, Runt-related transcription factor 2; ERK, Extracellular-regulated protein kinase; BMP, Bone Morphogenetic Protein; ALP, alkaline phosphatase; NF-кB, nuclear factor kappa-B; VEGFA, vascular endothelial growth factor-A; YAP, Yes-associated protein; OCN, osteocalcin; ECM, extracellular matrix; COX-2, cyclooxygenase; (MC3T3-E1) cells, mouse embryo osteoblast precursor; TAZ, transcriptional co-activator with PDZ-binding motif; ROCK, Rho associated protein kinase.

Stimulus type	Pathways	Mechanism	Effect	Reference
Axial compression	Activating Wnt signaling	Inhibiting the expression of DKK1	Upregulating the expression of OPG	Holguin et al. (2016)
Hypergravity		Inhibiting the expression of DDK2/enhancing the phosphorylation of *β*-catenin	Upregulating RUNX2/osterix/ALP/osteocalcin/ALP	Kawao et al. (2020)
Stretch stress	Activating Notch Signaling Pathway	Activating the expression of Piezo1	Upregulating ALP/RUNX2/OCN/BSP/promoting the osteogenic differentiation	Wang et al. (2020a)
Cyclic stretch		Upregulating the expression of Notch1/Notch2	Upregulating the expression of RUNX2/PTGS2/FOS	Ziouti et al. (2019)
FSS	Activating ERK signaling pathway	Upregulating the expression of COX-2/cyclin E1, inhibiting caspase-3	Promoting the proliferation of MC3T3-E1 cells	
FSS		Downregulating the expression of miR-140-5p/KLF4, upregulating the expression of VEGFA	Promoting the proliferation of MC3T3-E1 cells	Wang et al. (2021d), Bin et al. (2016)
Stretch stimulation	Activating RhoA signaling pathway	Upregulating the expression of YAP/TAZ	Promoting the osteogenic differentiation	Dupont et al. (2011)
High ECM stiffness		Upregulating the expression of YAP/TAZ	Triggering F-actin polymerization	Wagh et al. (2021)
oscillatory fluid flow		Activating ROCKII	Upregulating the expression of RUNX2	Arnsdorf et al. (2009)
FSS		Activating ROCK	Opening up of mechano- and voltage-sensitive calcium channels	Gardinier et al. (2014)
Mechanical tensile strain	Activating NF-κB signaling pathway	Upregulating the expression of BMP-2/BMP-4	Upregulating the expression of ALP/OCN	Wang et al. (2015)

## 5 Conclusion

**FIGURE 1:**
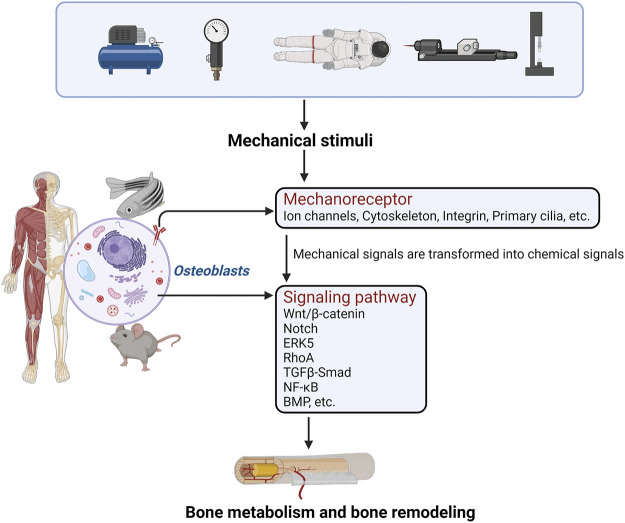
The perception of mechanical stress by osteoblasts first involves actions on mechanoreceptors through various pathways. Mechanoreceptors also regulate the expression of corresponding genes to convert mechanical stimulus signals into chemical stimuli, and regulate the relevant signal transduction pathways. Finally, bone metabolism and bone formation are regulated.

Loading and unloading mechanical stress affects the proliferation, differentiation, and function of osteoblast-like cells. Studies have shown that osteoblast-like cells, which are sensitive to mechanical stimulation, are the basic cell model for studying the processes of bone growth, development, and formation (Siddiqui and Partridge, 2016; Thomas and Jaganathan, 2021). The perception of mechanical stress by osteoblasts first involves actions on target genes through various pathways such as Ca^2+^, Piezo1, ECM-integrin-cytoskeleton, and cell regulatory factors. Mechanoreceptor also regulates the expression of corresponding genes to convert mechanical stimulus signals to chemical stimuli, and then regulates various receptors on the cell membrane, cytoplasm, and nucleus through chemoreceptors to regulate the bone formation mechanism (Steward and Kelly, 2015; Augat et al., 2021). (Figure 1).

Mechanical stimulation is an important regulatory factor for bone growth, reconstruction, and metabolism. Number experimental studies have investigated osteogenic effects under mechanical stress (Chermside-Scabbo et al., 2020; Eichholz et al., 2020; Jeon et al., 2021; Lei et al., 2021). The influence of different mechanical forces on bone tissue produces various effects. Analyzing the mechanism mediating the effects of mechanical stimulation on the regulation and metabolism of cell signaling pathways such as those of BMSCs, osteoblasts, osteoclasts, and osteocytes is currently a challenging research hotspot. Most previous studies used a single mechanical stimulus on a single mechanosensitive cell, and the mechanical loading methods and mechanical devices used did not have a relatively unified standard.

The results obtained from studies using randomly selected or combinations of different mechanical stimuli were no systematic and lacked standardization and accuracy. Consequently, designs of bone metabolism studies need to include a more suitable mechanical experimental environment to accurately control the mechanical parameters of mechanical stress stimulation, such as the type, duration, intensity, and cycle of mechanical stimulation). Furthermore, such studies should explore the influence of the body on mechanical stimulation. It is gratifying that mechanism-driven biology and biochemistry have been put into mathematical systems biology formats, this will certainly lead the research field to a more precise direction (Pastrama et al., 2018; Lavaill et al., 2020; Larcher and Scheiner, 2021).

This would provide an important theoretical basis for clinical treatment of bone diseases with a focus on issues such as bone health, fracture healing, and bone ingrowth at the bone-material interface. Further *in vivo* experiments need to study the changes in macroscopic bone tissue metabolic activity under stress stimulation, whereas future *in vitro* experiments should explore the specific mechanisms and signal transduction pathways mediating the effects of different mechanical stimuli on various mechanosensitive cells of bone tissue. These results could be used as a foundation for future orthopedics, stomatology, and tissue engineering research studies to advance the development of this discipline.
